# Supplementary material for: The genome sequence of Red Underwing, Catocala nupta Linnaeus, 1767
Source: Wellcome Open Res. 2025 Jan 30;10:36. [Version 1] doi: 10.12688/wellcomeopenres.23621.1 (PMC11851005; doi:10.12688/wellcomeopenres.23621.1)
Supplement: Peer review history

# Reviewer report: Reviewer response for version 1

**DOI:** 10.21956/wellcomeopenres.26061.r118072

**Published:** 2025-02-24

**Authors:** Arun Arumugaperumal

**Affiliations:** 1 Department of Biotechnology, Rajalakshmi Engineering College, Chennai, Tamilnadu, India

This is the first report of the whole genome sequence of the Red Underwing *Catocala nupta*. The genome size was 930.4 Mbp. The assembly reported here has 32 chromosomes. The mitochondrial genome of size 15.57 kb was also reported. The BUSCO analysis shows 99% completeness of genome sequence. The data note can be accepted for indexing.

Are sufficient details of methods and materials provided to allow replication by others?

Yes

Is the rationale for creating the dataset(s) clearly described?

Yes

Are the datasets clearly presented in a useable and accessible format?

Yes

Are the protocols appropriate and is the work technically sound?

Yes

Reviewer Expertise:

Bioinformatics, Genomics

I confirm that I have read this submission and believe that I have an appropriate level of expertise to confirm that it is of an acceptable scientific standard.

# Reviewer report: Reviewer response for version 1

**DOI:** 10.21956/wellcomeopenres.26061.r118070

**Published:** 2025-02-18

**Authors:** Maríndia Deprá

**Affiliations:** 1 Federal University of Rio Grande do Sul, Porto Alegre, Brazil

The article presents a robust and well-documented genomic assembly of a female specimen of *Catocala nupta*, following well-established protocols. The description of the methods is largely sufficient to allow replication of the study, with detailed information on the collection processes, DNA/RNA extraction, sequencing, and genome assembly. However, it would be beneficial to provide more details on the criteria for excluding haplotypic duplications and manual curation, and better clarify the parameters used in the assembly and annotation tools. Please, replace “male” by “female” in the end of the background section.

Are sufficient details of methods and materials provided to allow replication by others?

Partly

Is the rationale for creating the dataset(s) clearly described?

Yes

Are the datasets clearly presented in a useable and accessible format?

Yes

Are the protocols appropriate and is the work technically sound?

Yes

Reviewer Expertise:

genetics, DNA barcoding, molecular evolution

I confirm that I have read this submission and believe that I have an appropriate level of expertise to confirm that it is of an acceptable scientific standard.

# Reviewer report: Reviewer response for version 1

**DOI:** 10.21956/wellcomeopenres.26061.r118073

**Published:** 2025-02-04

**Authors:** Christophe Klopp

**Affiliations:** 1 INRAE, Castanet-Tolosan, France

The authors are presenting a first genome assembly, in chromosomes, of *Catocala nupta*. The article is well written and globally the methods are standards. 

 Comparing principal and alternative assembly sizes (930Mb vs 866Mb) shows that the authors are very close to haplotyped assemblies of a quite heterozygous individual. Therefore it would have been interesting to change strategy and provide the community with two haplotyped assemblies in chromosomes enabling to study heterozygocity patterns between haplotypes as well as to create the foundation of a species pangenome.

Are sufficient details of methods and materials provided to allow replication by others?

Partly

Is the rationale for creating the dataset(s) clearly described?

Yes

Are the datasets clearly presented in a useable and accessible format?

Partly

Are the protocols appropriate and is the work technically sound?

Partly

Reviewer Expertise:

Genome assembly and annotation

I confirm that I have read this submission and believe that I have an appropriate level of expertise to confirm that it is of an acceptable scientific standard, however I have significant reservations, as outlined above.
